# A comparative study of the inhibitory effects by caffeic acid, catechins and their related compounds on the generation of radicals in the reaction mixture of linoleic acid with iron ions

**DOI:** 10.3164/jcbn.16-54

**Published:** 2017-03-25

**Authors:** Yuji Matsui, Yoshie Tanaka, Hideo Iwahashi

**Affiliations:** 1 Department of Chemistry, Wakayama Medical University, 580 Mikazura, Wakayama 641-0011, Japan; 2 Wakayama Physical Therapy College, 229-2 Kitano, Wakayama 649-6331, Japan

**Keywords:** iron chelators, caffeic acid, chlorogenic acid, (–)-epicatechin, epigallocatechin

## Abstract

Caffeic acid and (+)-catechin, which are abundantly contained in coffee and tea, are typical polyphenols. In order to know the relative magnitudes of antioxidant activity, effects by caffeic acid, (+)-catechin and their derivatives on the formation of 4-POBN/carbon-centered linoleic acid-derived radical adducts were examined in the control reaction mixture of linoleic acid with FeCl_3_ at 30°C for 168 h. In the presence of 1.0 mM of the polyphenols, peak to peak heights of the third ESR signal resulted in 7.7 ± 2.4% (*n* = 3) (caffeic acid), 145 ± 13% (*n* = 3) (quinic acid), 4.4 ± 0.0% (*n* = 3) (chlorogenic acid), 104 ± 4.4% (*n* = 3) (ferulic acid), 4.3 ± 0.0% (*n* = 3) (noradrenaline), 12.5 ± 10.9% (*n* = 3) (gallic acid), 38.1 ± 7.1% (*n* = 3) [(+)-catechin], 47.9 ± 11.7% (*n* = 3) [(–)-epicatechin], 56.5 ± 1.6% (*n* = 3) (epigallocatechin), 13.5 ± 1.7% (*n* = 3) (catechol) and 83.7 ± 7.8% (*n* = 3) (resorcinol) of the control reaction mixture. All the compounds with catechol moiety exerted potent inhibitory effects on the radical formation except for (+)-catechin, (–)-epicatechin and epigallocatechin. (+)-Catechin, (–)-epicatechin and epigallocatechin may not exert the inhibitory effect as much possibly because they are less stable compared with caffeic acid. The resorcinol moiety in these molecules may also weaken their antioxidant activity.

## Introduction

Iron is present in the human body in great quantity in the form of heme and non-heme proteins. It plays a crucial role in electron transfer, cellular respiration, cell proliferation and differentiation, and regulation of gene expression.^(1)^ On the other hand, iron exposure is directly associated with the pathogenesis of many disorders, such as atherosclerosis, cancer and inflammation, possibly via the production of free radicals.^(2,3)^

Chlorogenic acid, caffeic acid (CA), noradrenaline and gallic acid are typical catechol compounds. Of the catechols, chlorogenic acid and CA are found naturally in various agricultural products such as coffee beans, potatoes, and apples.^(4,5)^ Chlorogenic acid is an ester of CA with quinic acid. Chlorogenic acid and CA have been known to be inhibitors of formation of hydroxyl radical in the reaction of 3-hydroxyanthranilic acid and hydrogen peroxide with ferric ions,^(6)^ lipid peroxidation and formation of lipid-derived radicals.^(7,8)^ Chlorogenic acid and CA also act as scavengers of superoxide, hydroxyl and peroxy radicals.^(9,10)^ It was reported that noradrenaline and dopamine provide an antioxidant defense in the brain against oxidatative stress.^(11)^ The chemically induced LDL oxidation is reduced by galloyl derivatives.^(12)^ Their antioxidative activities appear to be achieved through inhibiting the formation of the free radical by catechol moiety which has iron ion chelating activity.^(13)^

Catechins such as (+)-catechin, (–)-epicatechin (EC), epigallocatechin (EGC) and epigallocatechingallate (EGCG) are also typical catechol derivatives. They are tricyclic phenols (flavonoids) found in green tea. Catechins exert protective effects against oxidative damage of erythrocyte membrane,^(14)^ ethanol-induced fatty livers,^(15)^ cardiovascular diseases,^(16,17)^ inflammatory,^(18)^ and cancer.^(19)^ Catechins decreases 4-POBN/radical adducts formed in bile of rats after transplantation of ethanol-induced fatty livers.^(15)^

The radical scavenging activities of (+)-catechin and CA found in the two common beverages, coffee and tea, were investigated in detail in terms of their reaction with the stable radical 2,2-diphenyl-1-picrylhydrazyl in methanol.^(20,21)^ Meanwhile, the polyphenols are strong antioxidants due to their ability to chelate transition metals like iron as well as their radical scavenging activities.^(22)^ In order to examine the effect of their chelating ability on the formation of lipid-derived free radical in the reaction of linoleic acid with iron ions, we used electron spin resonance (ESR), high performance liquid chromatography-electron spin resonance (HPLC-ESR) and high performance liquid chromatography-electron spin resonance-mass spectrometries (HPLC-ESR-MS) and conducted the comparative study on CA and catechins in antioxidative activities.^(23)^

## Materials and Methods

### Chemicals

Caffeic acid, catechol, resorcinol, gallic acid, ferulic acid, quinic acid, chlorogenic acid, and (+)-catechin were purchased from Tokyo Kasei Kogyo, Ltd. (Tokyo, Japan). Ferric chloride (FeCl_3_) was from Wako Pure Chemical Industries, Ltd. (Tokyo, Japan). Linoleic acid and (–)-epicatechin were obtained from Sigma Aldrich Co. (St. Louis, MO). Noradrenaline was from Nacalai Tesque, Inc. (Tokyo, Japan). Epigallocatechin was purchased from Kishida Chemical Co., Ltd. (Osaka, Japan). Water used in these experiments was purified by passing through AUTOPURE WT101UV (Nihon Millipore Kogyo K.K., Yonezawa, Japan) after distillation.

### Control reaction mixture

In the control reaction mixture, there were 50 mM phosphate buffer (pH 7.4), 0.1 M 4-POBN, 0.89 mM linoleic acid, 0.38 M acetonitrile and 20 µM FeCl_3_ in a quartz test tube (100 mm long × 8 mm i.d.). 4-POBN is a spin-trapping agent. The reactions were performed at 30°C for 168 h. After the reaction, the reaction mixture was applied to the ESR (or HPLC-ESR or HPLC-ESR-MS).

### ESR studies

The ESR experiments were carried out on a JES-FR 30 Free Radical Monitor (JEOL Ltd., Tokyo, Japan). Operating conditions of the ESR spectrometer were: power, 4 mW; modulation width, 0.1 mT; time constant, 0.3 s. Magnetic fields were calculated by the splitting of MnO (ΔH_3–__4_ = 8.69 mT).

### HPLC-ESR chromatography

**Fig. 1:**
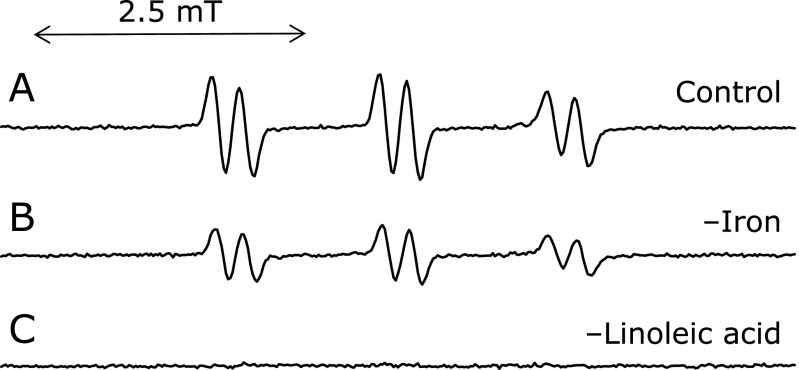
ESR Spectra of the control reaction mixtures. The reaction and ESR condition were as described in materials and methods section. In the control reaction mixture, there were 50 mM phosphate buffer (pH 7.4), 0.1 M 4-POBN, 0.89 mM linoleic acid, 0.38 M acetonitrile and 20 µM FeCl_3_. A, control reaction mixture. B, without 20 µM FeCl_3_. C, without 0.89 mM linoleic acid.

An HPLC used in the HPLC-ESR consisted of a model 7125 injector (Reodyne, Cotati, CA), a model L-7100 pump (Hitachi Ltd., Ibaraki, Japan). A semi-preparative column (300 mm long × 10 mm i.d.) packed with TSKgel ODS-120T (TOSOH Co., Tokyo, Japan) was used. Flow rate was 2.0 ml/min throughout the HPLC-ESR experiments. For the HPLC-ESR, two solvents were used: solvent A, 50 mM acetic acid; solvent B, 50 mM acetic acid/acetonitrile (20/80, v/v). A following combination of isocratic and linear gradient was used: 0–40 min, 100% A to 20% A (linear gradient); 40–60 min, 80% B (isocratic). The eluent was introduced into a model JES-FR30 Free Radical Monitor (JEOL Ltd.). The ESR spectrometer was connected to the HPLC with a Teflon tube, which passed through the center of the ESR cavity. The operating conditions of the ESR spectrometer were: power, 4 mW; modulation width, 0.2 mT; time constant, 1 s. The magnetic field was fixed at the third peak in the double-triplet ESR spectrum (α^N^ = 1.58 mT and α^H^β = 0.26 mT) of the 4-POBN radical adducts (Fig. 1).

### HPLC-ESR-MS chromatography

The HPLC and ESR conditions were as described in the HPLC-ESR. The mass spectrometer (MS) used in the HPLC-ESR-MS was a model M-1200 HS electrospray ionization (ESI)-MS (Hitachi Ltd.). The operating conditions of the ESI-MS were: nebulizer, 180°C; aperture1, 120°C; N_2_ controller pressure, 19.6 N/cm^2^; drift voltage, 70 V; multiplier, 2,000 V; needle voltage, 4,000 V; polarity, positive; resolution, 48. The mass spectra were obtained by introducing the eluent from the ESR detector into the ESI-MS system just before the peak was eluted. The flow kept at 50 µl/min while the eluent was introducing into the ESI-MS.

## Results and Discussion

### ESR Spectra of the control reaction mixtures

ESR spectrum of the control reaction mixture (without FeCl_3_ or linoleic acid) was measured (Fig. 1). A prominent ESR spectrum (α^N^ = 1.58 mT and α^H^β = 0.26 mT) was observed in the control reaction mixture (Fig. 1A). The ESR signals remained unchanged for the control reaction in the absence of light [90 ± 6% (*n* = 3) of the control reaction mixture], suggesting that light is not involved in the radical formation. For the reaction mixture without iron, the ESR signal decreased to 53 ± 3% (*n* = 3) of the control reaction mixture (Fig. 1B), suggesting that iron ions were involved in the radical formation. ESR peaks were hardly observed in the absence of linoleic acid (Fig. 1C). The result indicates that the radicals formed in the control reaction mixture are derived from linoleic acid.

### Time course of the ESR peak heights

**Fig. 2:**
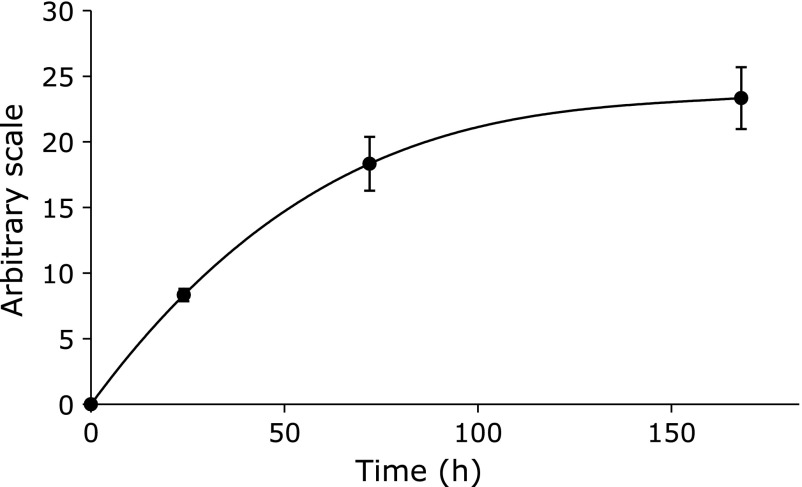
Time course of the ESR peak heights of control reaction mixture. The reaction and ESR condition were as described in materials and methods section except for reaction time. Reaction times were 0, 24, 72 and 168 h. The data represent the mean ± SD of independent three measurements. In the control reaction mixture, there were 50 mM phosphate buffer (pH 7.4), 0.1 M 4-POBN, 0.89 mM linoleic acid, 0.38 M acetonitrile and 20 µM FeCl_3_.

Time course experiments of the ESR peak height were performed for the control reaction mixture (Fig. 2). No ESR peak was observed at 0 h. The ESR peak height gradually increased and reached plateau at 168 h.

### HPLC-ESR analyses

**Fig. 3:**
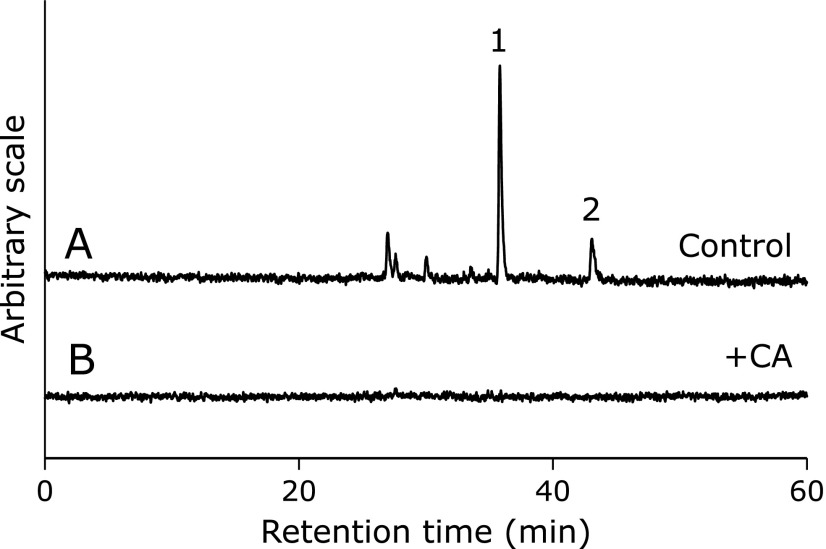
HPLC-ESR analyses. The reaction and HPLC-ESR conditions were as described in materials and methods section. In the control reaction mixture, there were 50 mM phosphate buffer (pH 7.4), 0.1 M 4-POBN, 0.89 mM linoleic acid, 0.38 M acetonitrile and 20 µM FeCl_3_. A, control reaction mixture. B, with 1 mM CA.

The HPLC-ESR analyses were performed for the control reaction mixture. On the HPLC-ESR elution profile of the control reaction mixture, two prominent peaks (peak 1 and peak 2) were observed at the retention times of 35.8 min (peak 1) and 43.1 min (peak 2) (Fig. 3A).

### HPLC-ESR-MS analyses of peaks 1 and 2

**Fig. 4:**
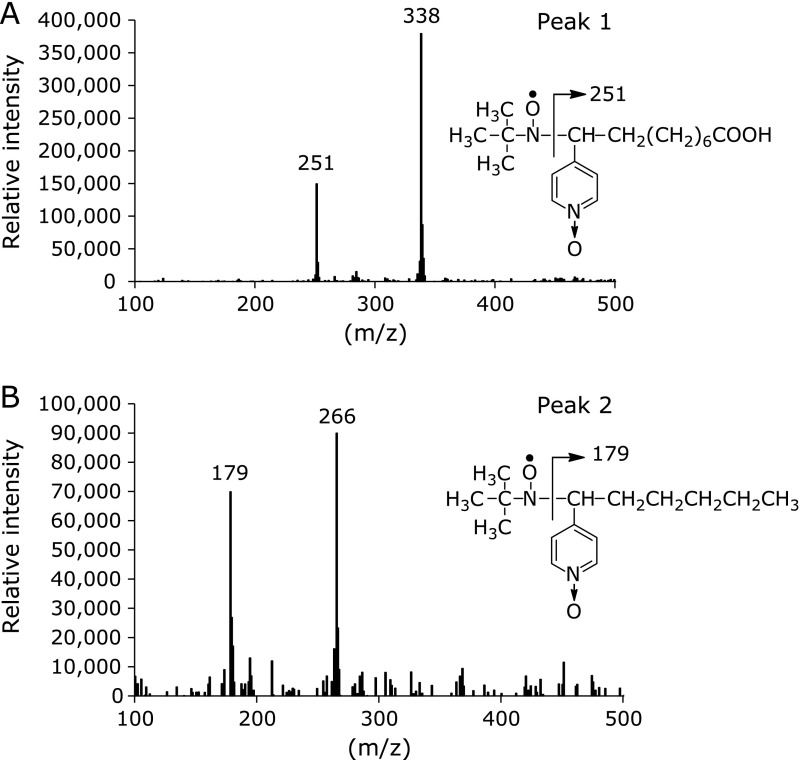
HPLC-ESR-MS analyses of the peak 1 and peak 2. The reaction and HPLC-ESR-MS conditions were as described in materials and methods section. In the control reaction mixture, there were 50 mM phosphate buffer (pH 7.4), 0.1 M 4-POBN, 0.89 mM linoleic acid, 0.38 M acetonitrile and 20 µM FeCl_3_. A, peak 1. B, peak 2.

In order to find out what kinds of radicals were formed in the control reaction mixture, HPLC-ESR-MS analyses were performed for peaks 1 and 2. Ions at m/z 251 and m/z 338 were observed in HPLC-ESR-MS analysis of the peak 1 (Fig. 4A), suggesting that peak 1 compound was 4-POBN/7-carboxyheptyl radical adduct. The ion m/z 338 corresponds to the protonated molecular ion of the 4-POBN/7-carboxyheptyl radical adduct, [M + H]^+^. A fragment ion at m/z 251 corresponds to the loss of [(CH_3_) _3_C(O)N] from the protonated molecular ion. HPLC-ESR-MS analysis of peak 2 gave ions at m/z 179 and m/z 266 (Fig. 4B), suggesting that peak 2 was 4-POBN/pentyl radical adduct. The ion m/z 266 corresponds to the protonated molecular ion of the 4-POBN/pentyl radical adduct, [M + H]^+^. A fragment ion at m/z 179 corresponds to the loss of [(CH_3_) _3_C(O)N] from the protonated molecular ion.

Our previous studies have also shown the formation of the 7-carboxyheptyl and pentyl radicals in the reaction mixture of linoleic acid with soya bean lipoxygenase and 13-hydroperoxyoctadeca-9,11-dienoic acid (13-HPODE) with ferrous ions (or cytochrome c or haematin).^(8,24–26)^

**Fig. 5:**
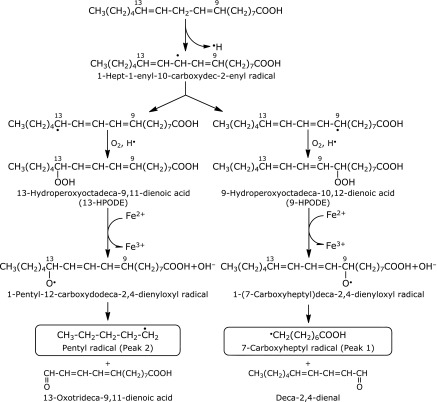
A possible reaction path for the formation of 7-carboxyheptyl radical (peak 1) and pentyl radical (peak 2) in the control reaction mixture.

We proposed a scheme to account for the formation of the 7-carboxyheptyl radical and pentyl radical (Fig. 5). As the ESR signal decreased to 53 ± 3% (*n* = 3) of the control reaction mixture for the reaction mixture without iron (Fig. 1B), iron complexes appear to catalyze the formation of 13-hydroperoxyoctadeca-9,11-dienoic acid (13-HPODE) and 9-hydroperoxyoctadeca-10,12-dienoic acid (9-HPODE) through the hydrogen atom abstraction at 11 carbon. Iron complexes such as iron(IV)-oxo and iron(III)-superoxo may initiate the O_2_-activation chemistry by abstraction of an H atom from the substrate.^(27,28)^ Product analysis and spin-trapping studies provided evidence for the formation of 1-pentyl-12-carboxydodeca-2,4-dienyloxyl radical and 1-(7-carboxyheptyl)deca-2,4-dienyloxyl radical through the reaction of 13-HPODE and 9-HPODE with ferrous ions.^(29–31)^ The ferrous ions may form in the following equilibrium to a small extent.

Fe^3+^ ⇄ Fe^2+^

The β scissions of 1-pentyl-12-carboxydodeca-2,4-dienyloxyl and 1-(7-carboxyheptyl)deca-2,4-dienyloxyl radicals resulted in the formation of pentyl radical and 7-carboxyheptyl radical.^(24–26,32–35)^ The pentyl radical could be a precursor of pentane.^(35)^

### Effect of CA on the reaction

**Fig. 6:**
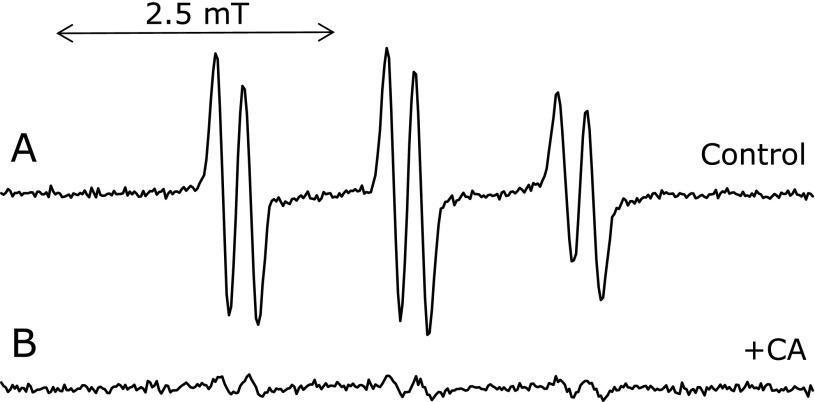
Effect of CA on the ESR spectra of the control reaction mixture. The reaction and ESR conditions were as described in materials and methods section. In the control reaction mixture, there were 50 mM phosphate buffer (pH 7.4), 0.1 M 4-POBN, 0.89 mM linoleic acid, 0.38 M acetonitrile and 20 µM FeCl_3_. A, control reaction mixture. B, control reaction mixture with 1 mM CA.

An ESR spectrum was measured for the control reaction mixture with 1.0 mM CA (Fig. 6B). The ESR peak height sharply decreased to 7.7 ± 2.4% (*n* = 3) of the control reaction mixture on addition of 1 mM CA.

In order to understand the effect of CA on respective radical formation, the control reaction mixture and control reaction mixture with 1.0 mM CA were analyzed using HPLC-ESR. On the HPLC-ESR elution profile of the control reaction mixture, two prominent peaks (peak 1 and peak 2) were observed at the retention times of 35.8 min (peak 1) and 43.1 min (peak 2) (Fig. 3A). The respective peaks disappeared when the control reaction mixture was added with 1 mM CA (Fig. 3B). Caffeic acid inhibited the formation of both radicals. Caffeic acid forms a chelate complex with iron ions.^(6)^ Therefore, the polyphenols possibly inhibit the following three steps (Fig. 5), i.e., step 1, the reaction of linoleic acid with iron complexes such as iron(IV)-oxo and iron(III)-superoxo to form 1-hept-1-enyl-10-carboxydec-2-enyl radical,^(27,28)^ step 2, the reaction between 13-HPODE and 1-pentyl-12-carboxydodeca-2,4-dienyloxyl radical, and step 3, the reaction between 9-HPODE and 1-(7-carboxyheptyl)deca-2,4-dienyloxyl radicals because iron ions participate in the three reactions. It has previously been shown that the step 2 is inhibited by the polyphenols.^(8)^

### Effect of CA on the reaction in the presence of EDTA

**Fig. 7:**
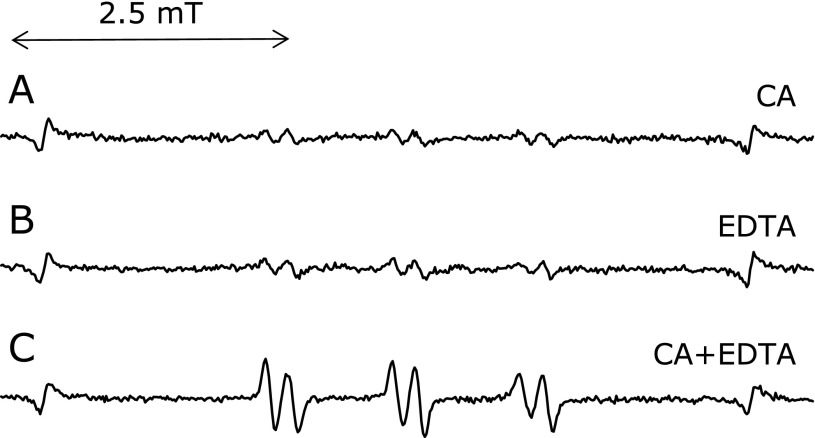
Effect of CA on an ESR spectra of the control reaction mixtures. The reaction and ESR conditions were as described in materials and methods section. In the control reaction mixture, there were 50 mM phosphate buffer (pH 7.4), 0.1 M 4-POBN, 0.89 mM linoleic acid, 0.38 M acetonitrile and 20 µM FeCl_3_. A, control reaction mixture with 1 mM CA. B, control reaction mixture with 1 mM EDTA. C, control reaction mixture with 1 mM CA and 1 mM EDTA.

ESR spectra were measured for the control reaction mixture with 1.0 mM CA in the presence of 1 mM EDTA (Fig. 7). On adding 1 mM CA, the ESR peak height sharply increased to 254 ± 21% (*n* = 3) of the control reaction mixture in the presence of EDTA. Caffeic acid enhanced the generation of the radicals in the presence of 1 mM EDTA. It has been reported that caffeic acid enhanced hydroxyl radicals and *t*-butylhydroperoxide-derived radicals in the presence of EDTA.^(6)^ The enhancement is possibly due to EDTA-ferric ion complexes being reduced by CA.

### Effect of several iron chelators on the reaction

**Fig. 8:**
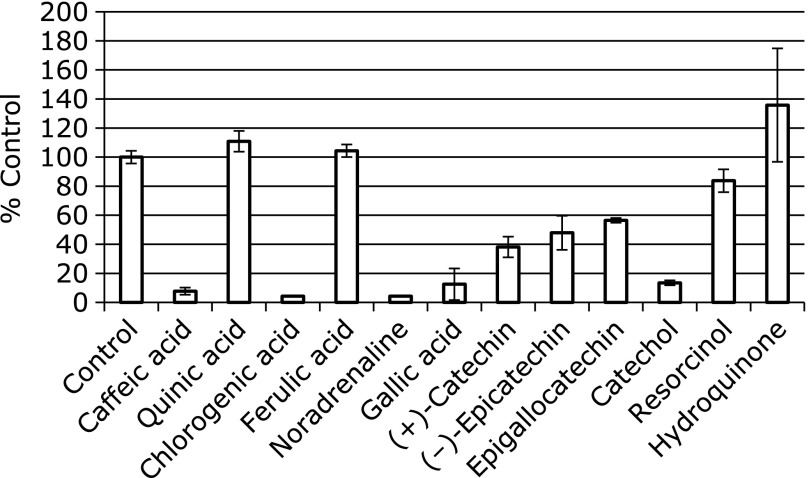
ESR peak heights of the control reaction mixtures with various iron chelators. The ESR spectra were observed for control reaction mixtures with 1 mM CA (or quininc acid, or chlorogenic acid, or ferulic acid, or noradrenaline, or gallic acid, or (+)-catechin, or (–)-epicatechin, or epigallocatechin, or catechol, or resorcinol, or hydroquinone). The reaction and ESR conditions were as described in materials and methods section. In the control reaction mixture, there were 50 mM phosphate buffer (pH 7.4), 0.1 M 4-POBN, 0.89 mM linoleic acid, 0.38 M acetonitrile and 20 µM FeCl_3_. The data represent the mean ± SD of independent three measurements.

In order to investigate the effects of several iron chelators on the radical formation, ESR spectra were measured for the control reaction mixture with 1 mM some iron chelators (Fig. 8). In the presence of the iron chelators, the peak heights of the ESR signals greatly decreased to 7.7 ± 2.4% (*n* = 3) (CA), 4.4 ± 0.0% (*n* = 3) (chlorogenic), 4.3 ± 0.0% (*n* = 3) (noradrenaline), 12.5 ± 10.9% (*n* = 3) (gallic acid), and 13.5 ± 1.7% (*n* = 3) (catechol) of the control reaction mixture, respectively. Meanwhile, the ESR peak heights decreased slightly to 38.1 ± 7.1% (*n* = 3) [(+)catechin], 47.9 ± 11.7% (*n* = 3) (epicatechin), 56.5 ± 1.6% (*n* = 3) (epigallocatechin). However, ESR peak heights were unchanged for the following compounds ; 110 ± 7.1% (*n* = 3) (quinic acid), 104 ± 4.3% (*n* = 3) (ferulic acid), 83.7 ± 7.8% (*n* = 3) (resorcinol) and 135 ± 39.0% (*n* = 3) (*p*-hydroquinone). The contribution of the direct radical-scavenging reaction of the phenolic compounds to the inhibitory effect seems to be small in these reactions. Of the compounds examined here, only compounds with catechol moiety, which were reported to be potent iron chelators,^(6)^ showed the inhibitory effect. Furthermore, caffeic acid enhanced the generation of the radicals in the presence of 1 mM EDTA. Thus, the inhibitory effects seem to be due to the chelation of iron ions. Polyphenols with catechol moiety also exerted inhibitory effects on radical formations in the other reactions through the chelation of ion ions.^(6,8)^ Since the three positional isomers of benzenediol, catechol, resorcinol and hydroquinone scavenged ^1^O_2_ to the same degree,^(36)^ ^1^O_2_ does not seem to be involved in the radical formation.

**Fig. 9:**
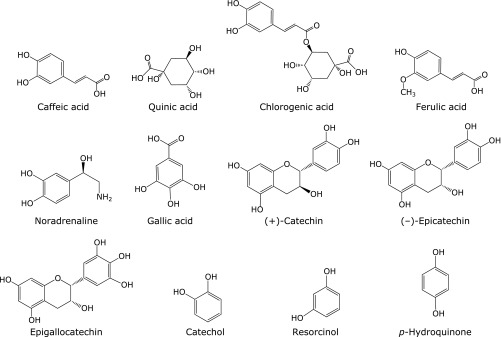
Chemical structures of compounds examined here.

**Fig. 10:**
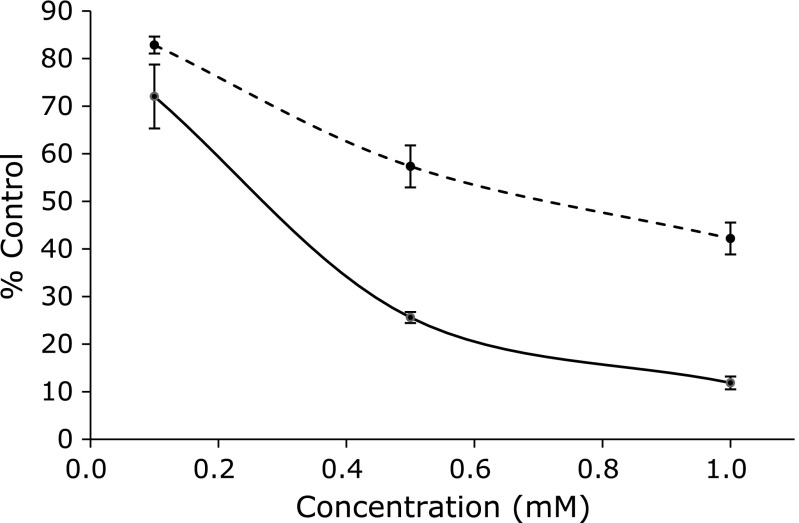
Inhibitory effects by CA and (–)-epicatechin on the radical formation in the reaction of linoleic acid with iron ion. The ESR spectra were observed for control reaction mixture with various concentrations of CA [or (–)-epicatechin]. In the control reaction mixture, there were 50 mM phosphate buffer (pH 7.4), 0.1 M 4-POBN, 0.89 mM linoleic acid, 0.38 M acetonitrile and 20 µM FeCl_3_. The reaction and ESR conditions were as described in materials and methods section. CA, solid line. (–)-Epicatechin, broken line. The data represent the mean ± SD of independent three measurements.

Caffeic acid, catechol, noradrenaline, chlorogenic, and gallic acid exert potent inhibitory effect on the formation of pentyl radical and 7-carboxyheptyl radical in the reaction of linoleic acid with iron ions. Of the compounds examined (Fig. 9), all these compounds, which exerted inhibitory effect on the formation of pentyl radical and 7-carboxyheptyl radical, have catechol moiety in the molecules. Interestingly, (+)-catechin, (–)-epicatechin, and epigallocatechin did not exert inhibitory effect on the formation of pentyl radical and 7-carboxyheptyl radical as much in spite of the catechol moiety in the molecules. That is also the case for the several different concentrations of (–)-epicatechin and CA (Fig. 10).

### Effects of (+)-catechin, (–)-epicatechin, and epigallocatechin on the reaction

**Fig. 11:**
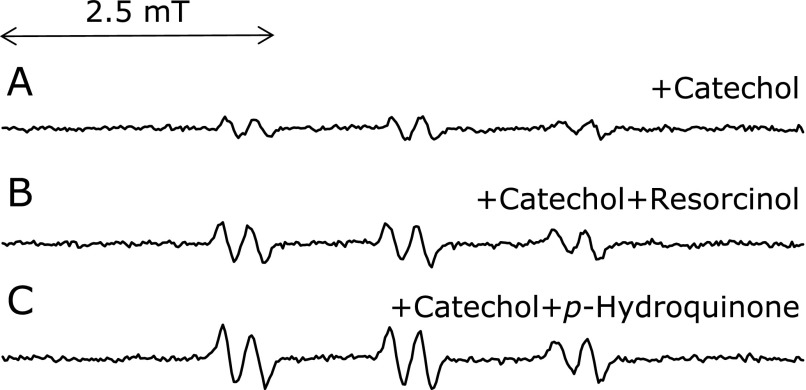
Effects of resorcinol (or *p*-hydroquinone) on the ESR peak height of the control reaction mixture with 1 mM catechol. Effects of resorcinol (or *p*-hydroquinone) on the ESR peak height of the control reaction mixture with 1 mM catechol were examined. The reaction and ESR conditions were as described in materials and methods section. In the control reaction mixture, there were 50 mM phosphate buffer (pH 7.4), 0.1 M 4-POBN, 0.89 mM linoleic acid, 0.38 M acetonitrile and 20 µM FeCl_3_. A, control reaction mixture with 1 mM catechol. B, control reaction mixture with 1 mM catechol in the presence of 1 mM resorcinol. C, control reaction mixture with 1 mM catechol in the presence of 1 mM p-hydroquinone.

In clarifying why (+)-catechin, (–)-epicatechin, and epigallocatechin did not exert the inhibitory effect as much on the radical formation, resorcinol moiety effect on the radical formation was examined. Addition of resorcinol to the control reaction mixture with catechol resulted in minimal enhancement of the radical formation (150 ± 24% of the control with catechol) (*n* = 3) (Fig. 11B). That was also the case for the addition of p-hydroquinone instead of resorcinol (182 ± 57% of the control with catechol) (*n* = 3) (Fig. 11C). The enhancement is presumably due to ferric ions being reduced by *p*-hydroquinone or resorcinol.

Stability of (–)-epicatechin was compared with CA. We determined the quantity of the remaining (–)-epicatechin (or CA) after 24 h incubation of control reaction mixture at 30°C. Caffeic acid and (–)-epicatechin decreased to 73 ± 9% (*n* = 3) (CA) and 62 ± 7% (*n* = 3) [(–)-epicatechin] of the initial concentration, respectively. (–)-Epicatechin appears to be a little unstable compared with CA.

Thus, (+)-catechin, (–)-epicatechin, and epigallocatechin cannot exert the inhibitory effect as much potentially because it has less stability and resorcinol moieties of the catechins.
